# It’s a moth! It’s a butterfly! It’s the complete mitochondrial genome of the American moth-butterfly *Macrosoma conifera* (Warren, 1897) (Insecta: Lepidoptera: Hedylidae)!

**DOI:** 10.1080/23802359.2020.1831991

**Published:** 2020-10-27

**Authors:** Bonnie S. McCullagh, Mackenzie R. Alexiuk, Josephine E. Payment, Rayna V. Hamilton, Melanie M. L. Lalonde, Jeffrey M. Marcus

**Affiliations:** Department of Biological Sciences, University of Manitoba, Winnipeg, MB, Canada

**Keywords:** Illumina sequencing, mitogenomics, Papilionoidea, Hesperiidae, Hedylidae

## Abstract

The taxonomic placement of the moth-butterfly, *Macrosoma conifera* (Warren 1897) (Lepidoptera: Hedylidae), has been controversial. The 15,344 bp complete *M. conifera* circular mitogenome, assembled by genome skimming, consists of 81.7% AT nucleotides, 22 tRNAs, 13 protein-coding genes, 2 rRNAs and a control region in the typical butterfly gene order. *Macrosoma conifera COX1* features an atypical CGA start codon while *ATP6, COX1, COX2,* and *ND5* exhibit incomplete stop codons completed by the post-transcriptional addition of 3′ A residues. Phylogenetic reconstruction places *M. conifera* as sister to the skippers (Hesperiidae), which is consistent with several recent phylogenetic analyses.

*Macrosoma conifera* (Warren 1897) is a moth-butterfly species (Lepidoptera: Hedylidae) found in Central and South America (Kawahara et al. 2018). It has been most intensively studied in Costa Rica, where its primary larval host plant was identified as *Conostegia xalapensis* (Melastomataceae), but it has also been observed to feed on *Miconia argentea*, *M. impetiolaris*, *M. smaragdina* (Melastomataceae), *Apeiba membranacea* and *Luehea speciosa* (Malvaceae) (Quesada 2019). *Macrosoma conifera* is known for its moth-like features such as nocturnal activity, clubless antennae and dark-adapted visual systems, which distinguish it from the predominantly diurnal butterfly families (Yack et al. 2007; Kawahara et al. 2018).

There has been considerable controversy regarding the relationship of the moth-butterflies to true butterflies and skippers (Scoble and Aiello 1990). At various times, *Macrosoma* has been associated with moth families Pyralidae and Geometridae; with butterfly families Papilionidae, Pieridae, and Nymphalidae; with skipper family Hesperiidae; and has been proposed as the sister taxon to all skippers and butterflies. These taxonomic placements have been based on either morphological characters (Kendall 1976; Scoble 1986; Weintraub and Miller 1987; Ackery et al. 1999; Kristensen and Skalski 1999) or phylogenetic analysis of limited numbers of molecular characters (Weller and Pashley 1995). More recent molecular phylogenetic analyses have supported a sister relationship between *Macrosoma* (Hedylidae) and skippers (Hesperiidae) (Wahlberg et al. 2005; Regier et al. 2009; Mutanen et al. 2010; Heikkilä et al. 2012; Kawahara et al. 2018). Here we report the complete mitochondrial genome sequence of *M. conifera* from specimen Mcon2013.1, collected in Braulio Carillo National Park, Heredia Province, Costa Rica (GPS 10.1519 N, 84.0945 W) on 2 May 2013 that has been pinned, spread, and deposited in the Wallis Roughley Museum of Entomology, University of Manitoba (voucher WRME0507732).

DNA was prepared (McCullagh and Marcus 2015) and sequenced by Illumina NovaSeq6000 (San Diego, California) (Marcus 2018). The sequencing library was prepared using NEBNext Ultra II DNA Library Prep Kit for Illumina (New England Biolabs, Ipswich, Massachusetts). The mitogenome of *M. conifera* (Genbank MT852025) was assembled by Geneious 10.0.9 from 23,184,240 paired 150 bp reads (Genbank SRA PRJNA662440) using a *Mallika jacksoni* reference mitogenome (Lepidoptera: Nymphalidae, MT704828 (Alexiuk et al. 2020)). Annotation was in reference to *M. jacksoni* (Lepidoptera: Nymphalidae, MT704828), *Kallimoides rumia* (Lepidoptera: Nymphalidae, MT704827 (Payment et al. 2020)), and *Junonia stygia* (Lepidoptera: Nymphalidae, MN623383 (Living Prairie Mitogenomics Consortium 2020)) mitogenomes. The *M. conifera* nuclear rRNA repeat (Genbank MT878224) was assembled to a *Coeliades ramanatek* reference sequence (Genbank MT859413, SRA SRX6097006 (Zhang et al. 2019)) and annotated using *C. ramanatek*, *Lerema accius* (Genbank MT859412, SRA SRX1085012 (Cong et al. 2015)) and *Pyrgus malvae* (Genbank MT859414, SRA SRX4091999, (Li et al. 2019)) (Lepidoptera: Hesperiidae) rRNA repeat sequences.

The *M. conifera* circular 15,344 bp mitogenome assembly was composed of 3,530 paired reads with nucleotide composition: 40.5% A, 10.9% C, 7.5% G, and 41.2% T. The *M. conifera* mitogenome gene order and composition is identical to the typical lepidopteran gene arrangement, shared by all families suspected of being closely related to Hedylidae (Park et al. 2016). Ten *M. conifera* mitochondrial protein-coding genes begin with typical ATG or ATT start codons, with the remaining genes beginning with ATA (*ND2*), ATC (*ND6*), or CGA (*COX1*) start codons (Liao et al. 2010). The mitogenome contains three protein-coding genes (*COX1, COX2, ND5*) with single-nucleotide (T) stop codons, and one protein-coding gene (*ATP6*) with a two-nucleotide (TA) stop codon completed by post-transcriptional addition of 3′ A residues. The locations and structures of tRNAs were determined using ARWEN v.1.2 (Laslett and Canback 2008). tRNAs have typical cloverleaf secondary structures except for trnS (AGN) where the dihydrouridine arm is replaced by a loop, while the mitochondrial rRNAs and control region are typical for Lepidoptera (McCullagh and Marcus 2015).

We reconstructed a phylogeny using complete mitogenomes from *M. conifera*, 50 additional ditrysian Lepidoptera mitogenomes, and a partial mitogenome sequence from basal lepidopteran *Micropterix calthella* (Lepidoptera: Micropterigidae) as an outgroup (Timmermans et al. 2014; Cong and Grishin 2016; Lalonde and Marcus 2017). Mitogenome sequences were aligned in CLUSTAL Omega (Sievers et al. 2011), then analyzed by both parsimony and maximum likelihood (model selected by jModeltest 2.1.7 (Darriba et al. 2012), followed by a likelihood ratio test (Huelsenbeck and Rannala 1997)) in PAUP* 4.0b8/4.0d78 (Swofford 2002) (Figure 1). Phylogenetic analysis places the swallowtail butterflies (family Papilionidae) as the basal lineage within the butterfly clade (superfamily Papilionoidea), followed by *M. conifera* and the skippers (family Hesperiidae) as sister taxa. This contradicts several early taxonomic hypotheses, but is consistent with several more recent molecular phylogenetic analyses (Wahlberg et al. 2005; Regier et al. 2009; Mutanen et al. 2010; Heikkilä et al. 2012; Kawahara et al. 2018). We conclude that the *Macrosoma* species in family Hedylidae fall within the butterfly clade. Since butterflies are a specialized clade of mostly diurnal moths (Kristensen 1999), it is also correct to refer to *Macrosoma* as moths.

**Figure 1.**
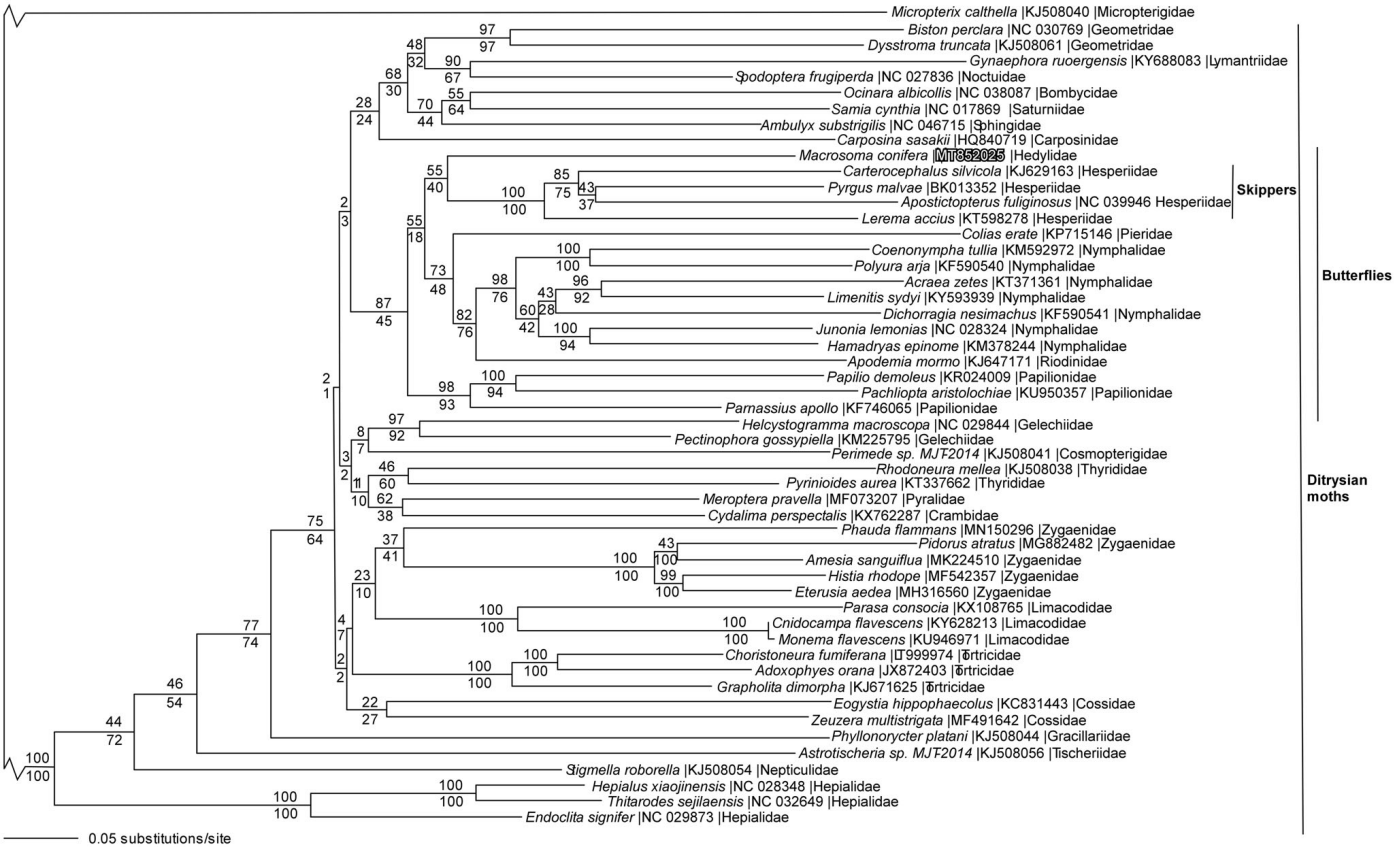
Maximum likelihood phylogeny (GTR + I + G model, I = 0.1590, G = 0.3930, likelihood score 316053.05651) of *Marcosoma conifera*, 50 additional ditrysian Lepidoptera mitogenomes (including the *Pyrgus malvae* (Hesperiidae) mitogenome (Genbank BK013352, SRA SRR7174492 (Li et al. 2019)), and *Micropterix calthella* (Micropterigidae)(Timmermans et al. 2014) as an outgroup based on 1 million random addition heuristic search replicates (with tree bisection and reconnection). One million maximum parsimony heuristic search replicates produced an identical tree topology (parsimony score 76038 steps). Numbers above each node are maximum likelihood bootstrap values and numbers below each node are maximum parsimony bootstrap values (each from 1 million random fast addition search replicates). Note that the very long branches leading to *Micropterix calthella* and the basal ditrysian moths are not drawn to scale to facilitate visualing the branching patterns within the ditrysian moths.

## Data Availability

The data that support the findings of this study are openly available in GenBank of NCBI at https://www.ncbi.nlm.nih.gov, reference numbers PRJNA662440, MT852025, MT859412-MT859414, MT878224, and BK013352.
